# A Highly Selective Biosensor with Nanomolar Sensitivity Based on Cytokinin Dehydrogenase

**DOI:** 10.1371/journal.pone.0090877

**Published:** 2014-03-04

**Authors:** Faming Tian, Marta Greplová, Ivo Frébort, Nicholas Dale, Richard Napier

**Affiliations:** 1 Warwick Biosensor Group, School of Life Sciences, University of Warwick, Coventry, United Kingdom; 2 Department of Molecular Biology, Centre of the Region Haná for Biotechnological and Agricultural Research, Palacký University, Olomouc, Czech Republic; University of Nottingham, United Kingdom

## Abstract

We have developed a *N* ^6^-dimethylallyladenine (cytokinin) dehydrogenase-based microbiosensor for real-time determination of the family of hormones known as cytokinins. Cytokinin dehydrogenase from *Zea mays* (*Zm*CKX1) was immobilised concurrently with electrodeposition of a silica gel film on the surface of a Pt microelectrode, which was further functionalized by free electron mediator 2,6-dichlorophenolindophenol (DCPIP) in supporting electrolyte to give a bioactive film capable of selective oxidative cleavage of the *N* ^6^- side chain of cytokinins. The rapid electron shuffling between freely diffusible DCPIP and the FAD redox group in *Zm*CKX1 endowed the microbiosensor with a fast response time of less than 10 s. The immobilised *Zm*CKX1 retained a high affinity for its preferred substrate *N* ^6^-(Δ^2^-isopentenyl) adenine (iP), and gave the miniaturized biosensor a large linear dynamic range from 10 nM to 10 µM, a detection limit of 3.9 nM and a high sensitivity to iP of 603.3 µAmM^−1^cm^−2^ (n = 4, R^2^ = 0.9999). Excellent selectivity was displayed for several other aliphatic cytokinins and their ribosides, including *N* ^6^-(Δ^2^-isopentenyl) adenine, *N* ^6^-(Δ^2^-isopentenyl) adenosine, *cis*-zeatin, *trans*-zeatin and *trans*-zeatin riboside. Aromatic cytokinins and metabolites such as cytokinin glucosides were generally poor substrates. The microbiosensors exhibited excellent stability in terms of pH and long-term storage and have been used successfully to determine low nanomolar cytokinin concentrations in tomato xylem sap exudates.

## Introduction

Plants are highly sensitive to concentration changes in naturally occurring cytokinins, with circulating concentrations of this plant hormone ranging from picomolar to several hundred nanomolar. Cytokinins are present in various forms, as free bases, ribosides and conjugates which contribute to a set of mobile signals which play a major role in the regulation of numerous physiological and developmental plant processes, including cell division, differentiation, root and shot growth and development [1], [2]. To understand better cytokinin biosynthesis, metabolism and mechanisms of regulation, quantitative methods for monitoring cytokinin concentrations are of great interest. Many highly sensitive analytical methods have been developed for detecting trace concentrations of cytokinins in crude plant extracts, including for instance high performance liquid chromatography (HPLC) [3] and its combination with mass spectrometry (MS) [4]–[6]. Such methods are exquisitely sensitive and molecule-specific [7], [8], but results arrive long after the experiment is run.

Genetic reporters have proved highly instructive and are widely used for some plant hormones, most notably auxin [9]. A cytokinin-sensitive genetic reporter has been improved but has somewhat limited utility [10], [11]. In many areas of developmental biology electrochemical sensors have been proved highly instructive and early cytokinin electrodes have been reported [12], [13]. An electrochemical immunosensor based on a competitive immunoassay was also reported for measurement of *N* ^6^-(Δ^2^-isopentenyl) adenosine (iPR) [14]. Neither of these electrochemical methods have been adopted by the plant community, in part because of insufficient sensitivity. For example, the electrochemical immunosensor had a working range of 5–300 µg/ml (25–1500 µM) while endogenous cytokinin concentrations are nanomolar [15].

Enzyme based amperometric biosensors provide a simple, fast, selective, convenient and low cost analytical technique. Cytokinin dehydrogenase (CKX; EC 1.5.99.12) degrades cytokinins by oxidative cleavage of *N^6^*- side chain using FAD as the electron-donating cofactor [16]–[19]. An amperometric cytokinin biosensor utilizing CKX (*At*CKX2) has been reported [20]. The selectivity of CKX for *N* ^6^- cytokinins was retained, but the sensor's sensitivity was again low (5 micromolar), too low to make it useful for studying cytokinin dynamics without sample concentration. A more efficient electron mediator was sought in order to develop more sensitive CKX-based cytokinin biosensors.

2,6-dichlorophenolindophenol (DCPIP) is widely known as a photometric pH and redox indicator, and its membrane permeability makes it a popular redox coupling agent in assays of bacteria [21]–[23] and bacterial biofuel cells [24]. DCPIP modified electrodes have also been applied for amperometric determination of NADH [25]–[28] and ascorbic acid [29]. Importantly, DCPIP has been shown to be one of the best redox electron mediators for CKX, supporting high turnover rates [17].

**Figure 1:**
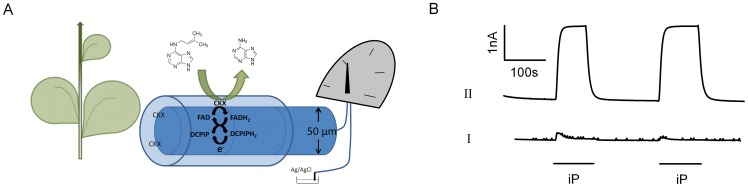
Construction of the cytokinin microbiosensor. (A) Diagrammatic representation of cytokinin microbiosensor. A silicate sol-gel entraps *Zm*CKX1 in a thin permeable layer around a platinum wire electrode. Cytokinins diffuse into the CKX layer where they are dehydrogenated by electron exchange with the FAD cofactor in CKX. The FAD is regenerated by DCPIP which cycles electrons with the electrode. (B) A cytokinin response is dependent on immobilised *Zm*CKX1. Response to 10 µM iP on microbiosensors using silica gel membranes doped without (I) and with *Zm*CKX1 (II) in a flow system with potassium phosphate buffer (pH 6.5) containing 10 µM DCPIP as supporting electrolyte. Operating potential, +400 mV.

In this paper, the high selectivity of cytokinin dehydrogenase has been coupled with the efficiency and mobility of DCPIP in order to develop a versatile, real-time microbiosensor for cytokinins. *Zm*CKX1 from *Zea mays* [30] has been adopted because it was shown to have higher catalytic efficiency than *At*CKX2 [31]. The enzyme was simultaneously electrodeposited and immobilised within a biochemically benign silica gel layer. At a fixed operating potential, the immobilised *Zm*CKX1 was driven by DCPIP to give continuous catalysis. Reduced mediator DCPIPH_2_ was oxidized at the surface of the microelectrode giving quantitative correlation between current and cytokinin concentration (Figure 1A). The performance of the sensor is described. The low nanomolar sensitivity of this microbiosensor is a 1000-fold improvement over the previous *At*CKX2 electrode and exemplifies the performance potential of dehydrogenases for microbiosensors.

## Materials and Methods

### Materials and Instrumentation

All chemicals were of analytical grade and used directly without any further purification. *N* ^6^-(Δ^2^-isopentenyl) adenine (iP), kinetin (K), *N* ^6^-(Δ^2^-isopentenyl) adenosine (iPR), *N* ^6^-(Δ^2^-isopentenyl) adenosine-5′-monophosphate (iPMP), *trans*-zeatin-9-glucoside (Z9G), *N* ^6^-(Δ^2^-isopentenyl) adenosine-7-*beta*-D-glucoside (iP7G), *N* ^6^-(Δ^2^-isopentenyl) adenosine-9-*beta*-D-glucoside (iP9G), (*RS*)-dihydrozeatin (DZ), *cis*-zeatin (cZ), *trans*-zeatin (Z), *trans*-zeatin riboside (ZR) and *cis*-zeatin riboside (cZR) were purchased from OlChemIm (Olomouc, Czech Republic). Thidiazuron (TDZ), gibberellic acid (GA), *N* ^6^-benzyladenine (*N* ^6^-BAP), abscisic acid (ABA) and adenosine (Ado) were from Sigma-Aldrich. All silanes including tetramethyl orthosilicate (TMOS), 3-glycidoxypropyl-dimethoxymethylsilane (GOPTMOS) and 3-aminopropyltrimethoxysilane (APTMOS) were also commercially obtained from Sigma-Aldrich.

Recombinant *Zm*CKX1 [16] was purified from the cell-free medium of *Pichia pastoris* X-33 transformed with pPICZ-A::*Zm*CKX1 (kind gift from K.D. Bilyeu). The yeast was grown overnight in BMGY medium (Invitrogen), then resuspended in YNB medium without amino acids (Difco) containing 0.1 M potassium phosphate buffer (pH 6.5), 0.4 µg/ml D-biotin and 0.5% (v/v) methanol and cultivated at 30°C with orbital shaking at 230 rpm. Additional methanol was added to 0.5% (v/v) at 24, 36, 48, 60 and 72 hours post-inoculation. Samples were harvested for assay of CKX activity in culture media. *Zm*CKX1 enzyme was finally purified by hydrophobic interaction chromatography on Octyl Sepharose and stored as a concentrated stock solution (15.2 mg/ml proteins) in 50 mM Tris buffer (pH 8.0) at −20°C. Potassium phosphate buffer solution (10 mM, pH 6.5, containing 0.1 M KCl) containing 10 µM DCPIP was prepared and used as common supporting electrolyte in amperometric detection experiments unless specified otherwise. As a common endogenous cytokinin, iP was employed as the standard substrate. All aqueous solutions were prepared with 18.2 MΩ deionized water.

A CHI 660B workstation (CH instruments) was used in amperometric experiments. A PG580 potentiostat–galvanostat (Uniscan instruments) was used for sol-gel electrodeposition. A three electrode cell equipped with a platinum foil counter electrode and a Ag/AgCl (saturated KCl) reference electrode was used for microbiosensor characterisation. Platinum microelectrodes with a diameter of 50 µm and a length of 0.5 mm were employed as the working electrode in all experiments.

### Preparation of cytokinin microbiosensor

*Zm*CKX1 was simultaneously immobilised onto a silica gel membrane-modified Pt microelectrode by electrodeposition under mild chemical conditions. This method has been well established and described previously [32], [33] In brief, silane precursors such as TMOS, GOPTMOS and APTMOS were pre-hydrolyzed with diluted HCl to the desired concentration. Then they were mixed with 50 mM Tris buffer (pH 7.1) to neutralize their pH. In order to stabilize enzyme in the sol mixture, additives such as glycerol and polyethylene glycol were introduced into the mixture. Next, 5 µl *Zm*CKX1 was thoroughly mixed with 8 µl of the hydrolysed sol mix, and transferred into a small glass capillary, into which the Pt microelectrode together with a counter electrode and a reference electrode were carefully inserted. The electrode was cathodically electroreduced under potentiostatic conditions between −0.9 and −1.2 V for 20 s. A transparent, smooth and robust gel layer doped with cytokinin dehydrogenase was formed uniformly around the Pt wire. Null sensors were prepared following the same procedure except that no enzyme was mixed with hydrolysed sol solution.

L-ascorbate oxidase (AOx) modified cytokinin microbiosensors were prepared by deposition of a further gel layer on top of a *Zm*CKX1-fabricated Pt electrode. The sol mix contained 20 units of AOx in 10 µl. Corresponding null sensors were prepared from the same AOx/sol mix on Pt electrodes.

The fabricated cytokinin microbiosensors were stored in 10 mM phosphate buffer pH 7.4 when not in use. For long term storage the cytokinin microbiosensors were dried and stored at 4°C.

### Collection of tomato plant root exudates sap

Tomato plants (*Solanum lycopersicum cv.* Espero) were potted at 8 weeks into Levington compost plus sand in 17 cm pots and grown to establish them for between 2–4 weeks in a temperature-controlled glasshouse (22°C, 16 h daylength). Plants were kept well watered. For collection of root pressure xylem sap exudates, plants were decapitated above the first leaf node. Sap flowed freely for up to 24 hours. The first drops were collected and discarded to remove the contribution of broken cells. Thereafter, sap was collected by pipette for use with the biosensor in a flow cell at room temperature, or frozen on dry ice and stored at −80°C. Transit to Palacký University in Olomouc, Czech Republic, was by courier on dry ice.

### Cytokinin determination by mass spectrometry

Sample (*ca* 200 µl) extraction and purification for endogenous cytokinin analysis was performed according to previous reports [6], [7]. The CK levels were quantified by ultra high performance liquid chromatography–electrospray tandem mass spectrometry (UHPLC-MS/MS) [7].

## Results and Discussion

### Optimising the cytokinin microbiosensor

Enzyme immobilization methods affect the analytical performance of amperometric biosensors and chemically mild immobilization conditions are desirable. Recently, electrodeposited silica sol-gel materials have proved very promising [34], [35]. In this method, at a sufficient cathodic potential, OH^−^ can be generated at the surface of an electrode. As long as the enzyme can withstand a period of elevated pH, a robust silica gel is evenly coated on the surface of Pt microelectrode. Using a solution of *Zm*CKX1 and hydrolysed silane sol a cytokinin microbiosensor was fabricated. A silica gel membrane-modified Pt microelectrode entrapped with *Zm*CKX1 (biosensor) and without (null sensor) showed an amperometric response to 10 µM iP in a flow system using potassium phosphate buffer (pH 6.5) containing 10 µM DCPIP as supporting electrolyte (Figure 1B). Compared with the flat recording trace (I) obtained on the null sensor, the biosensor displayed a sensing current of 2.16 nA to 10 µM iP (II). DCPIP was found to act as an active electron mediator for *Zm*CKX1 rather than directly catalysing dehydrogenation or oxidation of iP.

**Figure 2:**
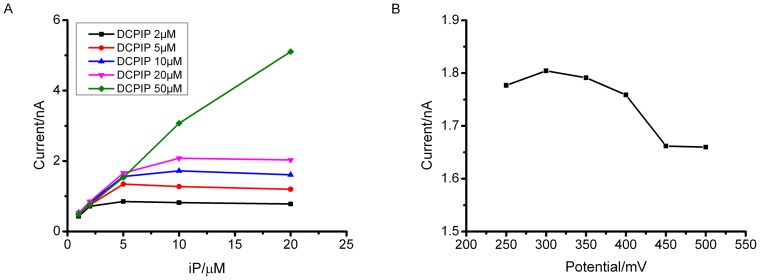
Optimum conditions of cytokinin microbiosensor. (A) Dependence of microbiosensor response on the concentration of DCPIP. Operating potential: +300 mV; (B) Response to 5 µM iP at different operating potentials in phosphate buffer (pH 6.5) containing 10 µM DCPIP as supporting electrolyte.

The analytical performance of *Zm*CKX1-based cytokinin microbiosensors could be highly reliant on the concentration of electron mediator and so the dependence on DCPIP was investigated across a concentration range of 2–50 µM in phosphate buffer (pH 6.5) and for a range of iP concentrations (Figure 2A). High concentrations of DCPIP provided better electron mediation at iP concentrations higher than 5 µM. But the cytokinin microbiosensor was little affected by DCPIP concentration at low iP concentrations (smaller than 5 µM). It is noted that the background current of the microbiosensors correlates with DCPIP concentration in phosphate buffer. Therefore, in order to maximise signal over noise, and taking account the low endogenous cytokinin concentrations likely to be recorded *in planta*, 10 µM DCPIP was adopted in the following experiments as electron mediator for the microbiosensor.

The performance of a mediated microbiosensor also relies greatly on operational potential. The response of the cytokinin microbiosensor to 5 µM iP at operating potentials from 250 to 400 mV (Figure 2B) showed optimal performance between 250 and 350 mV. When polarized at potentials lower than 300 mV it took a long time for the microbiosensor to achieve a steady background current due to less power for oxidizing DCPIPH_2_ in this operating potential range. Subsequent experiments were performed at 350 mV except where indicated in the figure captions.

### Cytokinin microbiosensor performance

**Figure 3:**
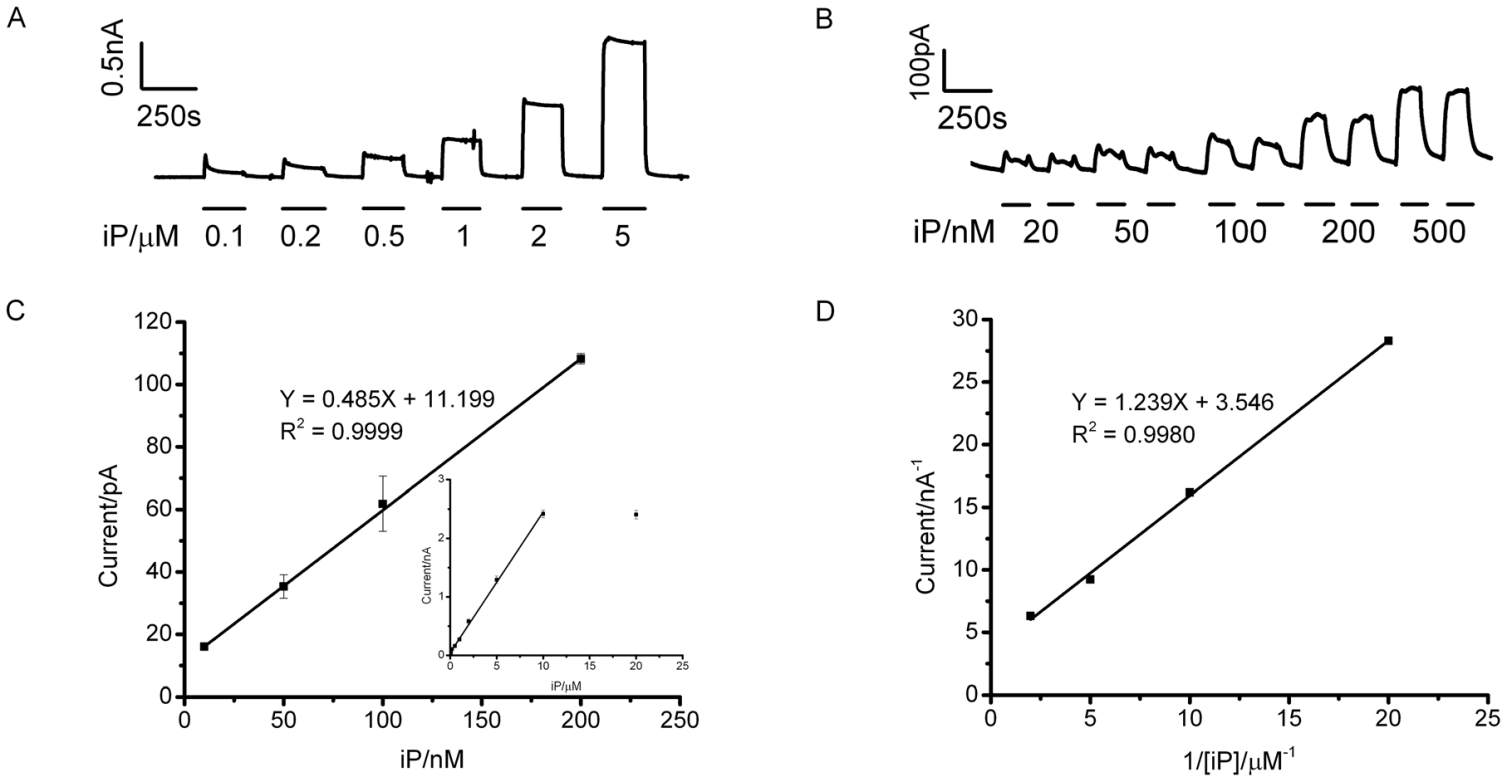
Performance of the cytokinin microbiosensor. (A) Amperometric response to iP at concentrations of 0.1, 0.2, 0.5, 1, 2 and 5 µM. Operating potential, +350 mV; (B) Amperometric response to iP at concentrations of 20, 50, 100, 200 and 500 nM. Operating potential, +350 mV; (C) Calibration plot of the microbiosensor at low iP concentrations ranging 10 to 200 nM, inset shows corresponding calibration over a larger concentration range; (D) Linear fit on a Lineweaver – Burk plot for a *Zm*CKX1-based cytokinin microbiosensor.

The responses of the cytokinin microbiosensor to different concentrations of substrate iP in phosphate buffer were recorded in a flow system (Figure 3A and 3B). The oxidation response increased linearly with iP concentration up to 5 µM. The cytokinin microbiosensor exhibited a rapid amperometric response, with a 10∼90% response time of 7.6±2.1 s (n = 15), which indicates fast and efficient electron mediation between DCPIP and immobilised *Zm*CKX1. Calibration was also measured over a lower concentration range of iP corresponding to a realistic range of cytokinin concentrations *in vivo* (Figure 3C). The inset of Figure 3C suggests the microbiosensor saturates above 10 µM iP. The data show that there was a linear dependence of amperometric current on iP concentration over the range 0.01∼10 µM, with a high sensitivity of 603.3±1.9 µAmM^−^1cm^−^2 (n = 4, R^2^ = 0.9999). A detection limit of 3.9 nM was calculated according to the criterion of 3 times the standard deviation of the amperometric signals from the substrate at the lowest concentration of the calibration plot divided by the sensitivity of the microbiosensor.

The apparent Michaelis-Menten constant (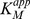), which characterizes affinity of iP for the immobilised *Zm*CKX1 can be obtained from a Lineweaver – Burk plot:
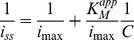
where *i_max_* and *i_ss_* are the amperometric current measured under substrate saturation and the steady-state current for a given substrate concentration (*C*), respectively. From the linear fit of such a plot (Figure 3D), the apparent *i_max_* and 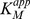 of the microbiosensor for iP were determined to be 0.28 nA and 0.35 µM. This apparent 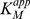 corresponds well with that reported for free *Zm*CKX1 in solution [36] suggesting that enzymatic activity of *Zm*CKX1 was well retained within the silica gel layer. Considering the two-electron transfer process for oxidation of DCPIPH_2_ on Pt electrodes, *i_max_* could be converted to *V_max_* with units of moles per second giving an estimate of the apparent *V_max_* of the microbiosensor for iP to be 1.45 fmol s^−1^.

### Selectivity and specificity of cytokinin microbiosensor

**Figure 4:**
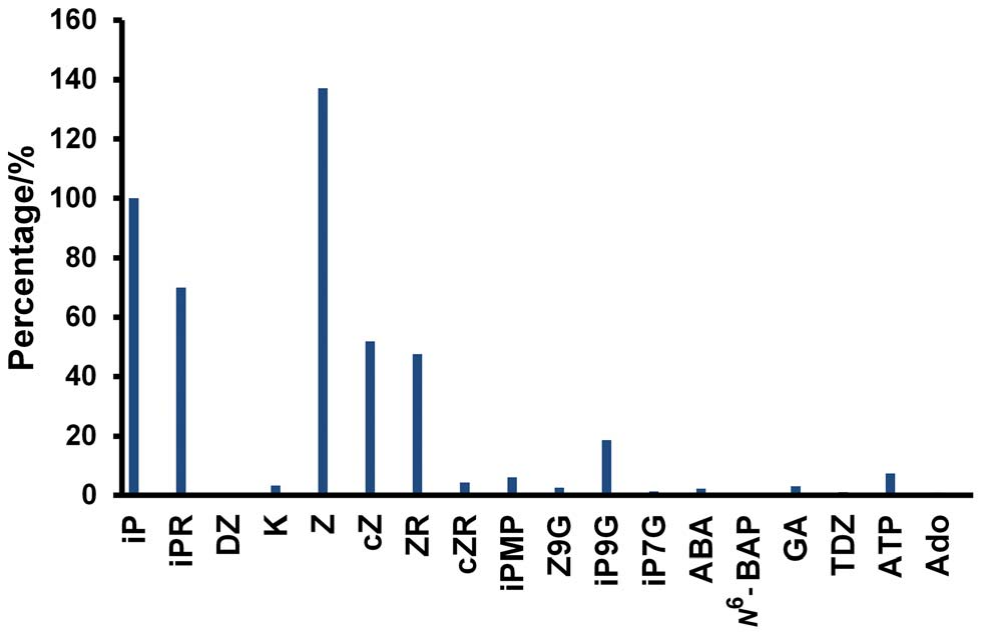
Selectivity of *Zm*CKX1-based cytokinin microbiosensor. Amperometric responses of cytokinins and analogues at 10 µM normalised to that of 10 µM iP. Operating potential, +350 mV. Key: *N* ^6^-(Δ^2^-isopentenyl) adenine (iP), kinetin (K), *N* ^6^-(Δ^2^-isopentenyl) adenosine (iPR), *N* ^6^-(Δ^2^-isopentenyl) adenosine-5′-monophosphate (iPMP), *trans*-zeatin-9-glucoside (Z9G), *N* ^6^-(Δ^2^-isopentenyl) adenosine-7-*beta*-D-glucoside (iP7G), *N* ^6^-(Δ^2^-isopentenyl) adenosine-9-*beta*-D-glucoside (iP9G), (*RS*)-dihydrozeatin (DZ), *cis*-zeatin (cZ), *trans*-zeatin (Z), *trans*-zeatin riboside (ZR) and *cis*-zeatin riboside (cZR), Thidiazuron (TDZ), gibberellic acid (GA), *N* ^6^-benzyladenine (*N* ^6^-BAP), abscisic acid (ABA) and adenosine (Ado).

Selectivity was investigated by monitoring amperometric responses from different cytokinins and analogues at a concentration of 10 µM. For ease of comparison, the responses were normalized to the response obtained from 10 µM iP (Figure 4). The aliphatic cytokinins and their ribosides were generally good substrates for the microbiosensor, including *trans*-zeatin, iP, iPR, *cis*-zeatin and *trans*-zeatin riboside. The most favorable substrate was *trans*-zeatin, 1.37 times that of iP. *Cis*-zeatin riboside was not a substrate, nor was the reduced dihydrozeatin. Aromatic cytokinins, K and *N^6^*-BAP, and the synthetic substituted urea cytokinin thidiazuron were also inactive, as were most cytokinin glucosides and the monophosphate iPMP. Plant hormones from other familes, abscisic acid and gibberellic acid were inactive along with the parental purine riboside adenosine. ATP showed a small response. Overall, the data for the cytokinin microbiosensor match almost exactly the substrate selectivity profile found previously using reaction rates measured using the continuous spectroscopic assay [30] and illustrate that the sensor will detect the most active endogenous cytokinins with high fidelity. The microbiosensor is shown to give rapid quantitation of (aliphatic) cytokinin concentrations. This output is an integrated cytokinin concentration that may be referred to as iP-equivalents. In this it differs from mass spectrometric analysis in that it cannot give concentrations for each contributory cytokinin.

### Stability of cytokinin microbiosensor

**Figure 5:**
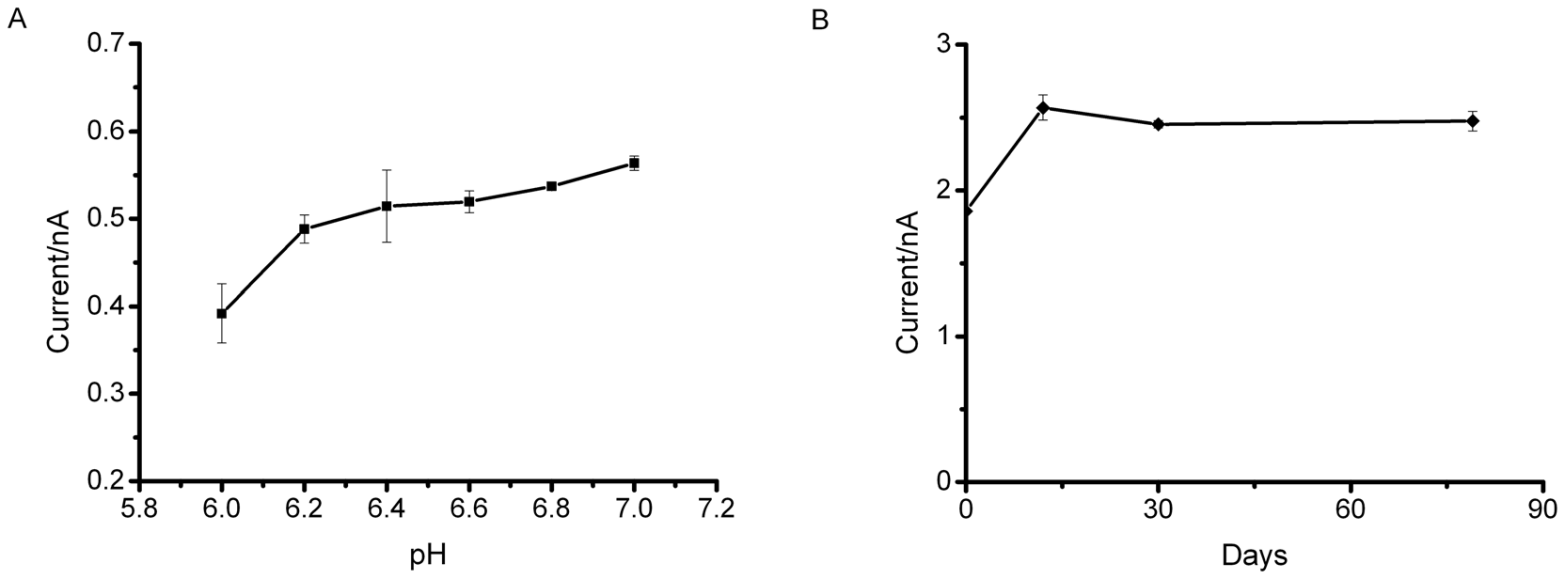
Stability of cytokinin microbiosensors. (A) Dependence of the response to 2 µM iP on pH using phosphate buffer at +300 mV. Each point is the mean obtained from 3 microbiosensors. (B) Long term storage stability of cytokinin microbiosensors. Each point represents the mean amperometric response to 10 µM iP from 4 microbiosensors at each different storage stage normalised to the response tested immediately after preparation (n = 6).

The dependence of the cytokinin microbiosensor on pH was studied by testing its response towards 2 µM iP in phosphate buffer with different pH (Figure 5A). As known, there is a two-proton process involved in electrochemical redox of DCPIP. Thus the redox potential would move 60 mV negatively for each pH unit. Operating potential was set at +300 mV for all measurements in order to minimize effect of pH on operating potential. The microbiosensor displayed good stability over pH range 6.2 to 7.0, which is appropriate for the mildly acidic range of plant sap.

The long term stability of the cytokinin microbiosensor was investigated by determining the amperometric response to 10 µM iP on a batch of microbiosensors prepared simultaneously (Figure 5B). Sensitivity was gained after an initial ageing process and activity was retained for over two months. This long term stability may arise from the mild electrodeposition conditions for silica gel formation as well as the biocompatible character of silica gel matrixes.

### Application of the cytokinin microbiosensor in tomato plant sap

**Figure 6:**
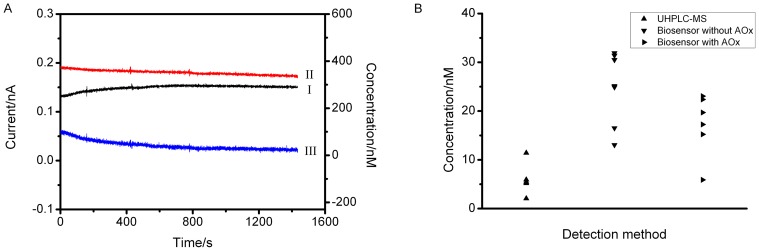
Cytokinin concentrations in xylem exudates. (A) Amperometric response curve of cytokinin microbiosensor recorded in tomato plant sap without (I) and with (II) DCPIP. Each trace represents difference between cytokinin microbiosensor and null sensor. The difference (II-I) represents cytokinin concentration (III) in the sap. Operating potential, +350 mV. (B) Comparison of cytokinin concentrations in sap determined by mass spectrometry UHPLC-MS (▴) and cytokinin microbiosensors prepared without (▾) and with (▸) L-ascorbate oxidase (AOx).

Tomato root-pressure exudates were collected from freshly decapitated, greenhouse-grown plants. The first drop of exudate was collected and discarded, after which sap was collected with a pipette. The sap was analysed on a multichannel potentiostat using a two electrode system equipped with a Ag/AgCl auxiliary electrode. A null sensor was paired with a cytokinin microbiosensor in order to deduct systematic differences such as background current generated by sap with and without DCPIP. Both sensor and null were polarized simultaneously at +350 mV in sap mixed without and with 10 µM DCPIP. The difference of current (microbiosensor minus null) obtained from each solution was plotted (Figure 6A). A current was recorded in each case, but a bigger oxidation current was generated in the presence of DCPIP. As *Zm*CKX1 does not catalyse cytokinin without the mediator, the difference in current may be related to the sensor calibrated with iP. According to the calibration (Figure 3C) the average concentration of cytokinin (iP equivalents) was found to be 24.8±7.5 nM (Figure 6B; n = 7).

Ascorbate is recognized as a frequent potential interference in biosensor records. The method of adopting electron mediator DCPIP should help to abate this concern for the cytokinin microbiosensor. More commonly, the enzyme L-ascorbate oxidase (AOx) is included in the sol, reducing the potential ascorbate signal by around a hundred-fold (Figure S1). Incorporating a sol layer of AOx around *Zm*CKX1 in our microbiosensors and using a null sensor prepared only with AOx, cytokinin measurements suggested little or no interference from ascorbate (Figure 6B) with an average concentration of cytokinin (iP equivalents) of 17.2±6.3 nM (n = 6). Equivalent sap samples were also frozen and sent for analysis by UHPLC-MS/MS [8] (Table S1; Figure 6B). Using data from Figure 4, the MS data were converted to iP equivalents to give cytokinin concentrations of 6.0±3.4 pmol/g (n = 5, Table S2), which is a little below that from cytokinin microbiosensors with or without AOx (between which there was no significant difference) (Figure 6B). All assessments confirmed that root exudate sap carries low nanomolar concentrations of the cytokinin family of plant growth regulators and the microbiosensor allows direct evaluation of cytokinin concentrations in real-time.

## Conclusions

We have demonstrated the development of *Zm*CKX1 based cytokinin microbiosensor. DCPIP freely diffuses into the porous silica gel film to exchange electrons rapidly with the FAD redox group of entrapped cytokinin dehydrogenase. The output response was fast, stable in less than 10 s and kinetic study indicated that the high affinity of *Zm*CKX1 for aliphatic cytokinins was retained. The cytokinin microbiosensor had a large linear dynamic range from 10 nM to 10 µM with a detection limit of 3.9 nM and a high sensitivity to iP of 603.3 µAmM^−1^cm^−2^. This performance is a thousand-fold improvement over previous cytokinin biosensors. By being able to resolve signals in the low nanomolar, the CKX biosensor is also more sensitive than previously described purine biosensors for adenosine and ATP based on oxidases [32], [37] and such nanomolar sensitivity is uncommon [38], [39]. The excellent specificity of free *Zm*CKX1 was also retained and the microbiosensor detected a range of cytokinins, including *N* ^6^-(Δ^2^-isopentenyl) adenine, *N* ^6^-(Δ^2^-isopentenyl) adenosine, *cis*-zeatin, *trans*-zeatin and *trans*-zeatin riboside. Due to dependence of its performance on DCPIP, the microbiosensor could be switched on/off by manipulating availability of the electron mediator. Furthermore, dehydrogenases have been used infrequently for biosensors and never yet with such sensitivity. This sensor electrochemistry can now be seen to open a rich, selective and sensitive toolkit.

The CKX microbiosensor was applied successfully to measure cytokinin concentrations in tomato plant sap. We show three sets of data, including LC/MS data, for comparison, all giving low nanomolar values (Figure 6). All the concentrations measured were somewhat lower than other records for tomato root xylem exudates (20 ng/ml; 100 nM) [40], nevertheless the somewhat lower values from the MS data merit further consideration. LC/MS is regarded as the ‘gold standard’ assay method by the cytokinin community. However, work-up includes an immunoaffinity clean-up step and it is possible this is not perfect despite corrections made by including reference standards [8]. Our biosensor requires no sample work-up and measurements were made directly on sample collection. One possible source of inaccuracy with electrochemical biosensors is ascorbic acid in the samples, but following standard procedures we showed this was not a significant contributor to the signal in this case (Figure 6B). The small difference in recorded concentration measurements between biosensor and LC/MS will require further experimentation, but the verification of results in the low nanomolar range indicates that CKX-based microbiosensors present a highly sensitive, rapid and accurate technique for determining cytokinin concentrations in plant extracts.

## Supporting Information

Figure S1 **Amperometric response to ascorbic acid (AA) with concentrations of 1, 10 and 100 µM added to null sensors prepared with and without L-ascorbate oxidase (AOx).** The running buffer (phosphate pH 6.5) included 10 µM DCPIP. Operating potential, +350 mV.
(TIF)

Table S1 **Cytokinin determinations by UHPLC-tandem mass spectrometry.** Data are shown for five independent samples (five plants) collected as for biosensor analysis. Abbreviations: <LOD, below the level of detection; OG, O-glucoside; 7G, 7-glucoside; 9G, 9-glucoside; 5′MP, 5′-monophosphate.
(DOCX)

Table S2 **Conversion of the mass spectrometry data from Table S1 into iP equivalents.** The comparative signal strength taken from Figure 4 is given as % iP equivalent (Conversion to iP /%). The measured concentration (Table S1) converted by this factor is given for each of the 5 sap samples. The sum of these values is the Total CKs given at the foot is the iP equivalent concentration.
(DOCX)
