# Supplementary figures and images for: A Highly Selective Biosensor with Nanomolar Sensitivity Based on Cytokinin Dehydrogenase
Source: PLoS One. 2014 Mar 4;9(3):e90877. doi: 10.1371/journal.pone.0090877 (PMC3942484; doi:10.1371/journal.pone.0090877)

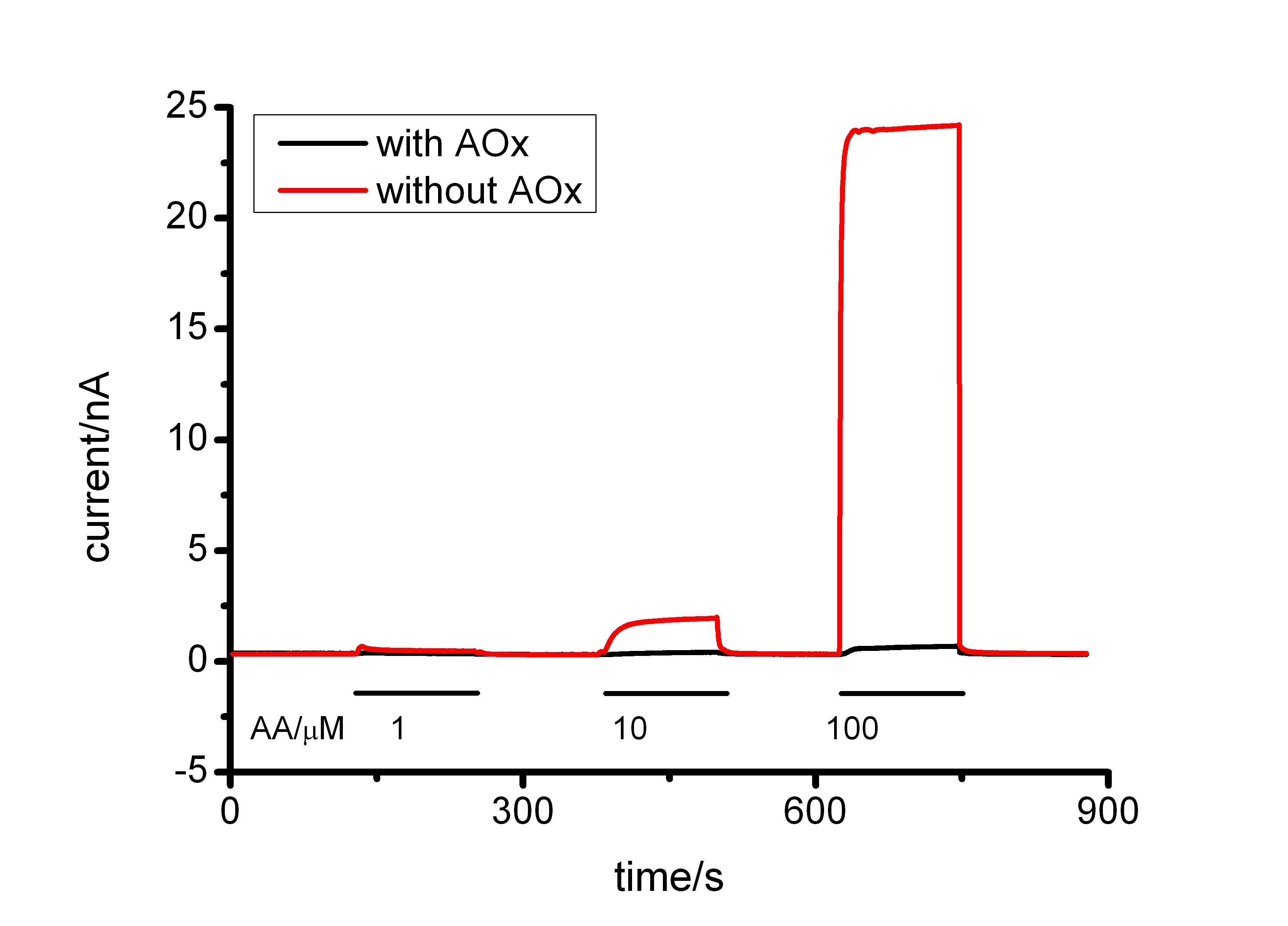

Supplement: Figure S1 — Amperometric response to ascorbic acid (AA) with concentrations of 1, 10 and 100 µM added to null sensors prepared with and without L-ascorbate oxidase (AOx). The running buffer (phosphate pH 6.5) included 10 µM DCPIP. Operating potential, +350 mV. (TIF) [file pone.0090877.s001.tif]
