# Supplementary material for: A Highly Selective Biosensor with Nanomolar Sensitivity Based on Cytokinin Dehydrogenase
Source: PLoS One. 2014 Mar 4;9(3):e90877. doi: 10.1371/journal.pone.0090877 (PMC3942484; doi:10.1371/journal.pone.0090877)
Supplement: Table S1 — Cytokinin determinations by UHPLC-tandem mass spectrometry. Data are shown for five independent samples (five plants) collected as for biosensor analysis. Abbreviations: <LOD, below the level of detection; OG, O-glucoside; 7G, 7-glucoside; 9G, 9-glucoside; 5′MP, 5′-monophosphate. (DOCX) [file pone.0090877.s002.docx]

**Table S1. Cytokinin determinations by UHPLC-tandem mass spectrometry.** Data are shown for five independent samples (five plants) collected as for biosensor analysis. Abbreviations: <LOD, below the level of detection; OG, O-glucoside; 7G, 7-glucoside; 9G, 9-glucoside; 5'MP, 5'-monophosphate.

| *Cytokinin levels in 1g of tomato xylem sap collected (pmol/g)* | | | | | |
| --- | --- | --- | --- | --- | --- |
|  | SAP-1 | SAP-2 | SAP-3 | SAP-4 | SAP-5 |
| *Weight (g)* | *0.1840* | *0.2959* | *0.2654* | *0.2770* | *0.2258* |
| *tZ* | 0.301 | 0.237 | 0.159 | 0.130 | 0.609 |
| *tZR* | 10.999 | 5.209 | 4.589 | 2.372 | 7.583 |
| *tZOG* | <LOD | <LOD | <LOD | <LOD | <LOD |
| *tZROG* | <LOD | <LOD | <LOD | <LOD | <LOD |
| *tZ7G* | 0.424 | 0.421 | 1.868 | 0.815 | 0.261 |
| *tZ9G* | <LOD | <LOD | <LOD | <LOD | <LOD |
| *tZR5'MP* | 0.017 | 0.011 | 0.008 | <LOD | <LOD |
| Total tZ-types | 11.741 | 5.877 | 6.624 | 3.317 | 8.453 |
| *cZ* | 0.057 | 0.052 | 0.046 | 0.023 | 0.057 |
| *cZR* | 5.151 | 2.112 | 2.057 | 0.900 | 2.022 |
| *cZOG* | <LOD | <LOD | <LOD | <LOD | <LOD |
| *cZROG* | <LOD | <LOD | <LOD | <LOD | <LOD |
| *cZ7G* | 0.088 | 0.297 | 0.856 | 0.519 | 0.129 |
| *cZ9G* | <LOD | <LOD | <LOD | <LOD | <LOD |
| *cZR5'MP* | <LOD | 0.008 | 0.025 | 0.033 | 0.028 |
| Total cZ-types | 5.297 | 2.469 | 2.985 | 1.476 | 2.235 |
| *DHZ* | 0.006 | 0.005 | 0.004 | 0.005 | 0.015 |
| *DHZR* | 0.172 | 0.079 | 0.091 | 0.155 | 0.434 |
| *DHZOG* | <LOD | <LOD | <LOD | <LOD | <LOD |
| *DHZROG* | <LOD | <LOD | <LOD | <LOD | <LOD |
| *DHZ7G* | 0.098 | 0.326 | 1.036 | 0.547 | 0.197 |
| *DHZ9G* | <LOD | <LOD | <LOD | <LOD | <LOD |
| *DHZR5'MP* | <LOD | <LOD | <LOD | <LOD | <LOD |
| Total DHZ-types | 0.277 | 0.409 | 1.131 | 0.706 | 0.646 |
| *iP* | 0.078 | 0.035 | 0.025 | 0.199 | 0.010 |
| *iPR* | 7.725 | 3.537 | 4.629 | 0.637 | 0.829 |
| *iP7G* | 2.651 | 2.583 | 10.714 | 4.895 | 2.031 |
| *iP9G* | <LOD | <LOD | <LOD | <LOD | <LOD |
| *iPR5'MP* | 0.038 | 0.061 | 0.072 | 0.011 | 0.004 |
| Total iP-type | 10.492 | 6.216 | 15.441 | 5.742 | 2.874 |
| *Total CKs* | *27.807* | *14.972* | *26.181* | *11.242* | *14.209* |
| Bases | 0.443 | 0.329 | 0.234 | 0.357 | 0.691 |
| Ribosides | 24.047 | 10.936 | 11.366 | 4.064 | 10.868 |
| Nucleotides | 0.055 | 0.081 | 0.106 | 0.044 | 0.032 |
| O-glucosides | *<LOD* | *<LOD* | *<LOD* | *<LOD* | *<LOD* |
| N-glucosides | 3.262 | 3.627 | 14.474 | 6.777 | 2.617 |
