# Supplementary material for: A Highly Selective Biosensor with Nanomolar Sensitivity Based on Cytokinin Dehydrogenase
Source: PLoS One. 2014 Mar 4;9(3):e90877. doi: 10.1371/journal.pone.0090877 (PMC3942484; doi:10.1371/journal.pone.0090877)
Supplement: Table S2 — Conversion of the mass spectrometry data from Table S1 into iP equivalents. The comparative signal strength taken from Figure 4 is given as % iP equivalent (Conversion to iP /%). The measured concentration (Table S1) converted by this factor is given for each of the 5 sap samples. The sum of these values is the Total CKs given at the foot is the iP equivalent concentration. (DOCX) [file pone.0090877.s003.docx]

**Table S2. Conversion of the mass spectrometry data from Table S1 into iP equivalents.** The comparative signal strength taken from Figure 4 is given as % iP equivalent (Conversion to iP /%). The measured concentration (Table S1) converted by this factor is given for each of the 5 sap samples. The sum of these values is the Total CKs given at the foot as the iP equivalent concentration.

|  | Conversion to iP /% | SAP-1 | SAP-2 | SAP-3 | SAP-4 | SAP-5 |
| --- | --- | --- | --- | --- | --- | --- |
| *tZ* | 137.15 | 0.41 | 0.32 | 0.22 | 0.18 | 0.84 |
| *tZR* | 47.48 | 5.22 | 2.47 | 2.18 | 1.13 | 3.60 |
| *tZOG* |  |  |  |  |  |  |
| *tZROG* |  |  |  |  |  |  |
| *tZ7G* |  |  |  |  |  |  |
| *tZ9G* | 2.59 |  |  |  |  |  |
| *tZR5'MP* |  |  |  |  |  |  |
| Total tZ-types |  | 5.64 | 2.80 | 2.40 | 1.31 | 4.44 |
| *cZ* | 51.78 | 0.03 | 0.03 | 0.02 | 0.01 | 0.03 |
| *cZR* | 4.37 | 0.23 | 0.09 | 0.09 | 0.04 | 0.09 |
| *cZOG* |  |  |  |  |  |  |
| *cZROG* |  |  |  |  |  |  |
| *cZ7G* |  |  |  |  |  |  |
| *cZ9G* |  |  |  |  |  |  |
| *cZR5'MP* |  |  |  |  |  |  |
| Total cZ-types |  | 0.25 | 0.12 | 0.11 | 0.05 | 0.12 |
| *DHZ* | 0.80 | 0.00 | 0.00 | 0.00 | 0.00 | 0.00 |
| *DHZR* |  |  |  |  |  |  |
| *DHZOG* |  |  |  |  |  |  |
| *DHZROG* |  |  |  |  |  |  |
| *DHZ7G* |  |  |  |  |  |  |
| *DHZ9G* |  |  |  |  |  |  |
| *DHZR5'MP* |  |  |  |  |  |  |
| Total DHZ-types |  | 0.00 | 0.00 | 0.00 | 0.00 | 0.00 |
| *iP* | 100.00 | 0.08 | 0.03 | 0.03 | 0.20 | 0.01 |
| *iPR* | 70.04 | 5.41 | 2.48 | 3.24 | 0.45 | 0.58 |
| *iP7G* | 1.35 | 0.04 | 0.03 | 0.14 | 0.07 | 0.03 |
| *iP9G* | 18.61 |  |  |  |  |  |
| *iPR5'MP* |  |  |  |  |  |  |
| Total iP-type |  | 5.52 | 2.55 | 3.41 | 0.71 | 0.62 |
| *Total CKs* |  | 11.41 | 5.46 | 5.92 | 2.07 | 5.17 |
